# Die Rolle Medizinischer Behandlungszentren für Erwachsene mit Behinderung in der ambulanten Versorgung – eine qualitative Fallstudie mit zwei MZEB

**DOI:** 10.1007/s00103-026-04265-z

**Published:** 2026-07-14

**Authors:** Thorsten Meyer-Feil, Jana Stucke, Cornelia Weiß

**Affiliations:** 1 Institut für Rehabilitationsmedizin, Universitätsmedizin Halle, Medizinische Fakultät der Martin-Luther-Universität Halle-Wittenberg, Profilzentrum Gesundheitswissenschaften, Magdeburger Straße 8, 06112 Halle (Saale), Deutschland; 2 Stiftungsprofessur für Medizinische Versorgung von Menschen mit Behinderung und chronischen Erkrankungen, Medizinische Fakultät OWL, Universität Bielefeld, Bielefeld, Nordrhein-Westfalen Deutschland

**Keywords:** Geistige Behinderung, Mehrfachbehinderung, Versorgungsforschung, Fall-Studie, Qualitative Studie, Intellectual disability, Multiple disabilites, Health services research, Case-study, Qualitative study

## Abstract

**Einleitung:**

Seit 2015 können Medizinische Behandlungszentren für Erwachsene mit geistiger Behinderung oder schweren Mehrfachbehinderungen (MZEB) zur spezialisierten Versorgung gegründet werden. Die MZEB entstehen im Spannungsfeld zwischen regulärer ambulanter Versorgung, die allen Menschen offenstehen sollte, und spezialisierter Behandlung. Die Zielgruppen wurden bislang nicht eindeutig definiert. Das vom Innovationsfonds geförderte Projekt MeZEB untersucht die Rolle der MZEB in der Versorgungspraxis.

**Methoden:**

Qualitative vergleichende Fallstudie im Zeitraum 2019–2022 an 2 MZEB mit Mixed-Methods-Design und qualitativer Hauptstudie. Zu 2 Erhebungszeitpunkten wurden Leitfadeninterviews mit Menschen mit Behinderung bzw. ihren An- und Zugehörigen geführt. Ergänzend erfolgten teilnehmende Beobachtungen, Gruppendiskussionen und Expert*inneninterviews.

**Ergebnisse:**

Die Nutzer*innen verbanden mit den MZEB hohe Erwartungen vor dem Hintergrund negativer Erfahrungen in der ambulanten Regelversorgung. Die Nutzung reichte von einmaligen Konsultationen bis zur langfristigen Begleitung. Eine wichtige Rolle spielten MZEB bei der Hilfs- und Heilmittelversorgung, sozialmedizinischen Diagnostik und Begutachtung. Sie boten zeitliche, organisatorische und emotionale Entlastung und hatten das Potenzial, die Versorgung grundlegend zu verbessern. Gleichzeitig zeigten sich ambivalente Bewertungen und weiterhin ungedeckte Versorgungsbedarfe.

**Diskussion:**

Die untersuchten MZEB unterschieden sich deutlich hinsichtlich Aufgabenprofil und Versorgungsansatz. Für Betroffene und Angehörige ist daher häufig unklar, welche Leistungen von einem MZEB erwartet werden können. Die Option, selber behandeln zu können, eine stärkere Orientierung an regionalen Versorgungsbedarfen sowie eine engere Kooperation der MZEB erscheinen sinnvoll.

## Abstract (en)

**Introduction:**

Since 2015, German social law has facilitated the founding of outpatient healthcare centres for adults with intellectual and multiple disabilities (MZEBs). MZEBs have emerged in a field characterized by tension between the need for an inclusive regular healthcare, which should be open to everyone, and special needs that must be provided in specialised centres. The target groups of MZEBs are still equivocal. One central aim of the research project called MeZEB funded by the German innovation fund was to get a better understanding of the role MZEBs play in healthcare based on daily care practice.

**Methods:**

Qualitative comparative case study of two MZEBs from 2019–2022 with a mixed-methods design and a qualitative main study, including open interviews at two timepoints with persons with disability or with their relatives or professional caregivers. Participatory observation of practice, focus groups with the MZEB team and expert interviews were also completed.

**Results:**

Users had high expectations of MZEBs due to negative experiences in outpatient standard care. Use of MZEBs showed substantial variation—from single consultations to long-term continuous use. MZEBs play an important role in providing access to special therapies and medical aids through interdisciplinary counselling and socio-medical diagnostics and assessment. MZEBs offered relief in terms of time, organisation and emotion and had the potential to improve individual care. At the same time, there are indications of ambivalent attitudes towards MZEBs as well as further unserved needs.

**Discussion:**

The examined MZEBs differed fundamentally in terms of their task profile and approach to care. Therefore, it follows that patients and their relatives are unclear of what services can be expected from an MZEB. The option of being able to treat patients themselves and having a stronger focus on regional care needs and closer cooperation between MZEBs seem sensible.

## Einleitung

Seit 2015 ermöglicht der § 119c des Sozialgesetzbuches 5 (SGB V) in Verbindung mit § 43c SGB V Medizinische Behandlungszentren für Erwachsene mit geistiger Behinderung oder schweren Mehrfachbehinderungen (MZEB) als Ergänzung für eine bedarfsgerechte Versorgung von erwachsenen Menschen mit Behinderung zu gründen. Schon in den 1990er-Jahren haben Fachverbände auf die besonderen Herausforderungen der medizinischen Versorgung von Menschen mit schweren Beeinträchtigungen hingewiesen [1]. Die Behindertenrechtskonvention der Vereinten Nationen (UN-BRK; [2]) fordert in ihrem Artikel 25 von den Vertragsstaaten u. a., dass sie eine „unentgeltliche oder erschwingliche Gesundheitsversorgung in derselben Bandbreite, von derselben Qualität und auf demselben Standard zur Verfügung [stellen] wie anderen Menschen“. Zudem sollen die Vertragsstaaten „Gesundheitsleistungen an[bieten], die von Menschen mit Behinderungen speziell wegen ihrer Behinderungen benötigt werden“, und zwar „so gemeindenah wie möglich …, auch in ländlichen Gebieten“.

Mit dem Rückenwind der UN-BRK wurden in Analogie zu den seit Ende der 1960er-Jahre entstandenen Sozialpädiatrischen Zentren (SPZ) MZEB als Teil der ambulanten Versorgung der Zielgruppe eingeführt, auch und gerade begründet durch Erfahrungen fehlender Möglichkeiten einer angemessenen Transition der Versorgung junger Volljähriger mit Behinderung von den SPZ in das Erwachsenenalter [3, 4]. Mit der Möglichkeit der Gründung der MZEB sollte auf ein Versorgungsdefizit reagiert werden, da im § 113c SGB V explizit darauf verwiesen wird, dass die „Ermächtigung … zu erteilen [ist], soweit und solange sie notwendig ist, um eine ausreichende Versorgung von Erwachsenen mit geistiger Behinderung oder schweren Mehrfachbehinderungen sicherzustellen“.

Damit ist in der UN-BRK und auch in der gesetzlichen Grundlage der MZEB in Deutschland ein grundlegendes Spannungsfeld angelegt: zwischen einer Versorgung, die im Sinne des gleichberechtigten Zugangs denen von Menschen ohne Behinderung entsprechen sollte, und der Anerkennung von besonderen Versorgungsbedarfen, die eine besondere Expertise und Versorgungsinstitutionen notwendig machen (vgl. [5]). Vor diesem Hintergrund ist es nicht verwunderlich, dass die Rolle der MZEB in der Versorgung in diesem Spannungsfeld gerade in der Einführungszeit der MZEB noch unbestimmt bleibt und sich erst noch in der Praxis herausbilden muss.

Diese unbestimmte Rolle der MZEB drückt sich u. a. als hoch relevantes Konfliktfeld in den Verhandlungen zu den Zulassungen von MZEB aus. So haben sich die Krankenkassen in den Verhandlungen mit den Leistungsanbietern auf ein internes, nie veröffentlichtes Eckpunktepapier der Krankenkassenverbände von 2016 bezogen, dessen Positionen in wesentlichen Punkten von den gesetzlichen Vorgaben abwichen [6]. Demnach hätten die MZEB keinen Behandlungsauftrag und sollten sich auf Diagnostik und eine Koordinations- bzw. Lotsenfunktion in der ambulanten Versorgung beschränken [6]. Dies steht in deutlichem Kontrast zur mutmaßlichen Intention des Gesetzgebers, der die MZEB explizit als *Behandlungszentrum* bezeichnet. Im § 119c, SGB V, Absatz 1, heißt es entsprechend: „Medizinische *Behandlungs*zentren für Erwachsene mit geistiger Behinderung oder schweren Mehrfachbehinderungen, die fachlich unter ständiger ärztlicher Leitung stehen …, können vom Zulassungsausschuss zur *ambulanten Behandlung* von Erwachsenen mit geistiger Behinderung oder schweren Mehrfachbehinderungen ermächtigt werden“ (Hervorhebung durch Autor*innen).

Die Zielgruppe der MZEB wurde ebenfalls über ein Versorgungsdefizit definiert, nämlich als „… diejenigen Erwachsenen …, die wegen der Art, Schwere oder Komplexität ihrer Behinderung auf die ambulante Behandlung in diesen Einrichtungen angewiesen sind“ (§ 119c, SGB V). Mit welchen Problemen sind Menschen mit Behinderung in der regulären Gesundheitsversorgung konfrontiert? Hierzu gehören u. a. fehlende bauliche Barrierefreiheit, eine Finanzierungslogik ambulanter Versorgung, die zeitaufwendige Betreuung von Patientinnen und Patienten „bestraft“, substanzielle Aus- und Weiterbildungsdefizite von Gesundheitsfachpersonal, ungedeckte sozialmedizinische Bedarfe von Menschen mit Behinderungen, Kommunikationsprobleme, praxiskompatible Fähigkeiten zur Kooperation bei den Untersuchungen, Angst und Verweigerung aufseiten der Betroffenen, eine erschwerte Einsicht in ärztliche Verordnungen, unklare Lokalisationen von Schmerzen, Unterstützungsbedarf für die Stellung von Anträgen, fehlende psychiatrisch-psychotherapeutische Angebote, fehlende Hilfsmittel, stressbedingte Verhaltensprobleme, vermeidbare Stressoren in der Versorgung, wie Zeitdruck, lange Wartezeiten, Lärm, sowie fehlende Personalkontinuität [3, 7, 8].

Allerdings wurde die Zielgruppe vonseiten der Krankenkassen nicht über Versorgungsbedarfe, sondern über präspezifizierte Diagnosegruppen und Grade der Behinderung > 70 bzw. ≥ 70 definiert, wie aus Berichten einzelner MZEB deutlich wird (vgl. [9, 10]), was grundlegende Kritik nach sich zog [8]. Die von der Bundesarbeitsgemeinschaft MZEB 2022 überarbeitete Rahmenkonzeption der MZEB hält im Sinne des SGB V daran fest, dass die Zielgruppe von MZEB Erwachsene mit geistiger oder schwerer Mehrfachbehinderung seien, die wegen der Art, Schwere oder Komplexität ihrer Behinderung auf die ambulante Behandlung im MZEB angewiesen sind [11] – unabhängig von Diagnosen oder anerkanntem Behinderungsgrad.

Auf der Homepage der Bundesarbeitsgemeinschaft MZEB (www. https://bagmzeb.de/mzeb-finden/) sind aktuell 65 MZEB aufgelistet, davon 54 als aktive MZEB. Diese Liste ist allerdings weiterhin deutlichen Veränderungen unterworfen und nicht komplett. Menschen mit Behinderungen und ihre Angehörigen berichten davon, Hunderte von Kilometern fahren mussten, um einen spezialisierten Behandler zu finden [7]. Neben einer regionalen Ungleichverteilung von MZEB kann dieser Umstand auch auf sehr unterschiedliche Spezialisierungen von MZEB verweisen. Für eine Abschätzung, inwieweit Bedarfe mit der vorhandenen Struktur gedeckt werden können, scheint es noch zu früh, da es an einer grundsätzlichen Festlegung von Bedarfskriterien fehlt.

MZEB zeichnen sich durch eine hohe Heterogenität ihrer fachlichen Schwerpunkte, Organisationsstruktur, Anbindung sowie personellen und sächlichen Ausstattung aus [6]. Gemeinsam sollte allen MZEB eine ärztliche Leitung und ein multiprofessionelles Team sein. Laut § 43c SGB V haben „Versicherte … Anspruch auf nichtärztliche Leistungen, insbesondere auf psychologische, therapeutische und psychosoziale Leistungen, wenn sie … durch ein medizinisches Behandlungszentrum nach § 119c erbracht werden und erforderlich sind, um eine Krankheit zum frühestmöglichen Zeitpunkt zu erkennen und einen Behandlungsplan aufzustellen. Dies umfasst auch die im Einzelfall erforderliche Koordinierung von Leistungen.“ Beratungsleistungen können u. a. durch Pflegefachkräfte, therapeutische Fachkräfte wie Physiotherapie, Ergotherapie, Logopädie und auch durch Fachkräfte der sozialen Arbeit durchgeführt werden. Zu den Leistungen der MZEB gehören ebenfalls eine interdisziplinäre sozialmedizinische Beurteilung und Begutachtung für alle Sozialleistungsträger sowie die Hilfsmittelversorgung [5].

MZEB sollen einen spezifischen Versorgungsbedarf erfüllen, der aktuell in der ambulanten Regelversorgung nicht bzw. nicht in angemessenen Umfang (s. oben UN-BRK, Art. 25 [2]) gedeckt werden kann. Dabei ist eine entsprechende Versorgungsinstitution zwar notwendig, sollte aber zugleich nicht als mögliche Ausweich- oder Verweisstelle für eine subjektiv überforderte ambulante Versorgung dienen. Vor diesem Hintergrund ist es nicht verwunderlich, dass weiterhin unklar ist, was – bei hohen Erwartungen – MZEB leisten sollen und können [8], und damit auch, welche Aufgaben und Rolle MZEB in der Versorgungspraxis tatsächlich ausfüllen.

Das übergreifende Ziel der vom Innovationsfonds geförderten Studie MeZEB (Versorgung von Erwachsenen mit geistiger Behinderung oder schweren Mehrfachbehinderungen vor und nach Einführung von Medizinischen Zentren (MZEB), Förderkennzeichen 01VSF18040) bestand in der Beschreibung und Analyse der Unterschiede der medizinischen Versorgung von Erwachsenen mit geistiger Behinderung oder schweren Mehrfachbehinderungen durch die Einführung von MZEB im Vergleich zur Standardversorgung. Die 3 inhaltlichen Fragestellungen lauteten:

1. Wie ist die aktuelle medizinische Versorgung von Menschen mit geistigen oder Mehrfachbehinderungen?
2. Wie verändert sich diese Versorgung im Zuge der Einführung von MZEB mit Behinderung?
3. Welche Empfehlungen zur Weiterentwicklung der Versorgung im Rahmen der gesetzlichen Krankenversicherung lassen sich ableiten?

Vor dem Hintergrund der oben genannten Spannungsfelder zielt die Ergebnisdarstellung darauf, ein besseres Verständnis für die Aufgaben und die Rolle von MZEB in der Praxis der Gesundheitsversorgung zu erhalten.

## Methoden

**Abb. 1:**
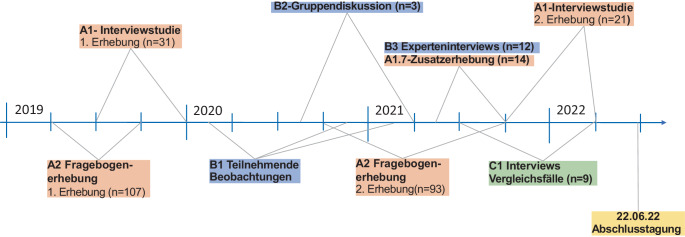
Arbeitspakete und Ablauf des Projekts MeZEB. Die Teilprojekte sind in Tab. 1 dargestellt

Bei MeZEB handelt es sich um eine Mixed-Methods-Studie mit einer qualitativen Hauptstudie. Die Darstellung des kompletten Designs findet sich bei Weiß et al. [12]. Sie wurde von 2019 bis 2022 an der Universität Bielefeld durchgeführt. Eine Übersicht über die Arbeitspakete der Studie findet sich in Abb. 1. Ein Teilprojekt von MeZEB (A1) rekonstruierte die Versorgungserfahrungen von Menschen mit Behinderung vor Eintritt in das MZEB und ca. 24 Monate später (Tab. 1). Dabei wurden im Rahmen eines Leitfadeninterviews nach Möglichkeit die Menschen mit Behinderung selbst befragt; ergänzend oder alternativ wurden ihre An- bzw. Zugehörigen befragt bzw. einbezogen. Ebenso kam ein standardisierter Fragebogen mit einem Fokus auf Versorgung an 2 Erhebungszeitpunkten zum Einsatz. In 2 MZEB aus 2 benachbarten Bundesländern (Niedersachsen und Nordrhein-Westfalen) wurden begleitende Beobachtungen, Gruppendiskussionen mit den MZEB-Teams sowie Expert*inneninterviews mit Mitarbeitenden durchgeführt. Die einzelnen methodischen Zugänge und ihre jeweiligen Instrumente sind bei Weiß et al. [12] beschrieben. Zusätzlich wurde in einem weiteren Teilprojekt die Versorgung in einer Vergleichsregion im Rahmen einer Interviewstudie mit Vergleichsfällen, d. h. Patient*innen ohne MZEB-Versorgung bzw. ihre An- und Zugehörigen, untersucht. Die Darstellung der Zitate aus den Interviews beinhaltet ein Pseudonym der interviewten Person und die Zeilenzahlen im jeweiligen Transkript des Interviews. Zu den Methoden gehört auch die Durchführung einer Abschlusstagung mit verschiedenen Stakeholdergruppen, um zentrale Ergebnisse des Projekts zu reflektieren und Handlungsempfehlungen abzuleiten.

**Tab. 1:** Teilprojekte des Projekts MeZEB

Teilprojekte	Methoden	Details
A1 Versorgungserfahrungen	Interviewstudie mit Nutzer*innen bzw. An- und Zugehörigen beider MZEB	1. Erhebungszeitpunkt, 21 Interviews zum 2. Erhebungszeitpunkt; im Rahmen der Zusatzerhebung A1.7 zu Auswirkungen der COVID-19-Pandemie auf die Versorgung 14 Interviews mit Nutzer*innen bzw. An- und Zugehörigen aus MZEB 1
A2 Versorgungsdokumentation	Fragebogenstudie Nutzer*innen oder An- und Zugehörige beider MZEB	107 Personen wurden zum 1. Erhebungszeitpunkt, 93 Personen zum 2. Erhebungszeitpunkt mit Fragebogen befragt
B1 Prozessabläufe MZEB	Teilnehmende Beobachtung Mitarbeiter*innen und Patient*innen	In beiden MZEB wurden mehrtägige Beobachtungen durchgeführt, u. a. Teilnahme an Versorgungskontakten
B2 Kooperation und Teamarbeit	Gruppendiskussionen Mitarbeiter*innen des Teams	In beiden MZEB wurden insgesamt 3 Gruppendiskussionen (MZEB 1: 2; MZEB 2: 1) durchgeführt
B3 Ergänzende Infos MZEB	Expert*inneninterviews Mitarbeiter*innen	In beiden MZEB wurden insgesamt 12 Expert*inneninterviews mit unterschiedlichen an der Versorgung beteiligten Gesundheitsprofessionen durchgeführt
C1 Versorgung in einer Vergleichsregion/Vergleichsfälle	Interviewstudie Vergleichsfälle Patient*innen ohne MZEB	Es wurden 9 Interviews mit Personen bzw. ihren An- und Zugehörigen, die kein MZEB nutzen, geführt
Abschlusstagung	Ergebnispräsentation, gemeinsame Diskussion, Ableitung von Handlungsempfehlungen	Die Ergebnisse des Projekts wurden vorgestellt und es wurden zusammen mit den 3 Stakeholdergruppen Betroffene, Professionelle und Wissenschaftler*innen Handlungsempfehlungen abgeleitet

Der Zugang zu den Teilnehmenden für die Leitfadeninterviews erfolgte über den o. g. Fragebogen (*n* = 107), der beim persönlichen Erstkontakt der betroffenen Person im MZEB ausgehändigt wurde und in dem die Bereitschaft zur Teilnahme an der Interviewstudie abgefragt wurde. Insgesamt hatten 76 Personen (MZEB 1: *n* = 40; MZEB 2: *n* = 36) ihre Bereitschaft zur Teilnahme erklärt. Die Auswahl der Fälle erfolgte im Sinne eines purposiven Samplings schrittweise nach den Prinzipien der minimalen und maximalen Kontrastierung von Merkmalen (Maximum-Variation-Sampling nach Alter, Geschlecht, Art und Schwere der Beeinträchtigung, Wohnsituation).

Zum 1. Erhebungszeitpunkt (07–12/2019) wurden 31 Interviews durchgeführt, davon 14 (45 %) mit den Patient*innen selbst (ggf. unter Beteiligung von bis zu 2 Angehörigen) und 17 (55 %) ausschließlich mit An- bzw. Zugehörigen. 15 (48 %) der Erstnutzer*innen waren weiblich, das Alter der Befragten lag zwischen 20 und 59 Jahren. Zum 2. Erhebungszeitpunkt (09/2021–03/2022) wurden 21 Interviews geführt, davon 9 (43 %) mit Patient*innen selbst und 12 (57 %) mit An- und Zugehörigen. 12 (57 %) der Interviewten waren weiblich und 9 (43 %) männlich. Das Alter der Teilnehmenden lag zwischen 22 und 61 Jahren. Zwischen beiden Erhebungen lagen nicht nur ca. 2 Jahre ambulante medizinische Versorgung, sondern auch allgemeine und grundlegende Veränderungen der Versorgungsgestaltung als Folge der Coronavirus(COVID-19)-Pandemie. Es wurden 3 Gruppendiskussionen mit insgesamt 16 Mitarbeitenden unter Beteiligung aller im jeweiligen MZEB beschäftigten Berufsgruppen durchgeführt sowie 12 individuelle Expert*innen-Interviews.

Das eine MZEB aus Niedersachsen repräsentiert einen traditionsreichen Standort mit einem Schwerpunkt in der orthopädischen Behandlung von Kindern, Jugendlichen und erwachsenen Menschen mit Behinderungen. Mit dem MZEB haben hier Erwachsene die Möglichkeit der Behandlung durch verschiedene Fachärzt*innen und Therapeut*innen unter „einem Dach“. Untersuchungen und Behandlungen fanden im Kontext des sozialen Umfelds einschließlich Beratung und Anleitung der Bezugspersonen statt. Über die Projektlaufzeit ist das MZEB kontinuierlich gewachsen und hatte Ende 2021 neue Räumlichkeiten bezogen. Es kamen Mitarbeiter*innen und (medizinische) Fachdisziplinen hinzu.

Das zweite MZEB aus Nordrhein-Westfalen (NRW) wurde an einem ebenso traditionsreichen Standort gegründet. Es verfügt über ein kleines engagiertes Team mit einem psychiatrischen Schwerpunkt. Die in NRW bestimmenden Rahmenbedingungen für MZEB, die die Behandlung von Patient*innen ausschloss, begünstigten die Rolle als Gesundheitskoordinator und Netzwerkpartner im Verbund mit den zuweisenden Haus- und Fachärzten. Die Spezialisten stimmten ihre Ergebnisse und Therapiekonzepte mit Angehörigen, Betreuer*innen, komplementären Einrichtungen sowie niedergelassenen Ärzt*innen und Therapeut*innen ab.

## Ergebnisse

### Erwartungen, Wünsche, Bedürfnisse

Die Praxis der MZEB wird durch die Vorstellungen und Erwartungen der verschiedenen Beteiligten geprägt, insbesondere jedoch durch die Bedürfnisse und Bedarfe der Patient*innen bzw. ihrer An- und Zugehörigen. Darüber hinaus beeinflussen auch die fachlichen Hintergründe und die beruflichen Erfahrungen der Mitarbeitenden die Ausgestaltung der Versorgung. Die befragten Nutzer*innen der MZEB schilderten in den Interviews ihre Wünsche bei Aufnahme ins MZEB und gaben damit Einblick in ihre bis dahin im Rahmen der ambulanten medizinischen Versorgung ungedeckten Versorgungsbedarfe und -bedürfnisse. Für sie war es normal, dauerhaft Schwierigkeiten beim Zugang zu Diagnostik, Behandlung und Vorsorge zu erleben. Die Perspektiven von Betroffenen, die für sich selbst sprechen konnten, und Angehörigen, die wir gleichsam stellvertretend befragten, waren dabei unterschiedlich. Die interviewten Personen mit Behinderung berichteten von Unsicherheiten, die sich durch Probleme in der Kommunikation und Interaktion mit Professionellen des medizinischen Personals zeigten. Regelhaft konnten durch die jeweilige Einschränkung bedingte spezielle Bedarfe und Bedürfnisse dieser ausgesprochen heterogenen Gruppe nicht berücksichtigt werden. Die Betroffenen wünschten sich in ihren individuellen Bedarfen und Bedürfnissen ernst genommen zu werden sowie eine bessere Versorgung durch vernetztes Arbeiten der an der Versorgung Beteiligten. Sie wünschten sich eine respektvolle Kommunikation mit ihnen und nicht über sie hinweg, eine feste „Anlaufstelle“ sowie die Bereitschaft, gemeinsam neue Lösungswege zu erproben („Experimentierfreude“).

Angehörige wünschten sich dagegen primär lokale Anlaufstellen und damit geringere Entfernungen zu Versorgungseinrichtungen sowie feste „Anlaufpunkte“ mit Zuständigkeit für die Patient*innen. Die an der Versorgung Beteiligten sollten die Patient*innen „kennen“, also auf längere Frist angelegte Versorgungsbeziehungen aufweisen. Erhofft wurden geringere Wartezeit auf und bei Terminen sowie Ärzt*innen, die über spezialisiertes Wissen und Erfahrung im Umgang mit Betroffenen verfügen. Patient*innen mit weiterreichenden Bedürfnissen und Anpassungsschwierigkeiten sollten nicht als Störfaktor gesehen werden. Die Praxen sollten nicht überfüllt, die an der Versorgung Beteiligten nicht überfordert sein. Es sollten angemessene Möglichkeiten und Durchhaltevermögen bei der Diagnostik und damit größere Handlungssicherheit in der Therapie und Versorgung bestehen.

Zum 1. Erhebungszeitpunkt berichteten die 31 Interviewteilnehmer*innen von einer Vielzahl ungedeckter Bedarfe und Bedürfnisse, die sie im MZEB zu decken suchten. Mit Eintritt ins MZEB brachten die Nutzer*innen und ihre (häufig aktiv beteiligten) Angehörigen daher eine globale Erwartungshaltung mit. Sie wünschten sich, in einer spezialisierten Einrichtung dauerhaft anzukommen und bedarfsgerecht versorgt zu werden. Die Teilnehmer*innen berichteten ausnahmslos von großen Hoffnungen und hohen Erwartungen, die sie mit Nutzung des MZEB verbänden. Diese bezogen sich vor allem auf erwartetes Fachwissen und Erfahrungen. Außerdem erhofften sie eine Anlaufstelle, bei der verschiedene Fachgebiete vernetzt arbeiten.

### Nutzungsmuster

Zum 2. Interview waren noch 21 von den ursprünglich 31 Befragten bereit. Nach unserem Kenntnisstand hatten die 10 Personen, die die Teilnahme an der Studie abbrachen, bereits vorher die Nutzung des jeweiligen MZEB beendet oder abgebrochen. Die Nutzungsmuster der verbliebenen 21 Betroffenen waren heterogen: 11 Teilnehmer*innen nahmen das MZEB weiterhin regelmäßig in Anspruch. Bei einer teilnehmenden Person wurde die Versorgung nach dem Wechsel der Wohneinrichtung beendet, da die aufnehmende Einrichtung keine Möglichkeit sah, die im MZEB begonnene Versorgung fortzusetzen. Die Angehörige einer Patientin berichtete, dass diese als „gesund entlassen“ wurde. Bei 6 weiteren Patient*innen bestand zum Zeitpunkt der Befragung kein weiterer Anlass, das MZEB aufzusuchen. Patient*innen sowie An- und Zugehörige berichteten in diesen Fällen, dass ihre Erwartungen an Diagnostik und Versorgung nicht erfüllt worden sind. Zwei weitere Angehörige berichteten, die Versorgung abgebrochen zu haben, waren aber zugleich verunsichert, da sie nicht einschätzen konnten, inwieweit alle diagnostischen und therapeutischen Möglichkeiten ausgeschöpft wurden.

Die Muster der Inanspruchnahmen zeigten sich heterogen. Die dauerhafte und regelmäßige Nutzung eines MZEB blieb einer Minderheit vorbehalten, die im jeweiligen MZEB eine passfähige Versorgung gefunden hatte. Sowohl in der Darstellung der Nutzer*innen als auch in der allgemeinen Darstellung aus Sicht der Mitarbeiter*innen reichte die Inanspruchnahme von einmaliger Konsultation bis zu langfristiger und regelmäßiger Nutzung, von hochspezialisierter gezielter Nachfrage einzelner Leistungen bis zu umfassender Beratung und Begleitung, von der Etablierung als Hauptansprechpartner*innen bis hin zur punktuellen Nutzung als Zusatzangebot. Unterschiede zwischen den beiden MZEB waren darin begründet, dass eines der beiden MZEB mit einem kleinen Mitarbeitendenstamm keine Behandlungsangebote machen konnte und sowohl für weiterführende spezialisierte Diagnostik als auch für Behandlungen an weitere Institutionen vermitteln musste. Dagegen konnte das andere MZEB Leistungen unter einem Dach anbieten und auch bestimmte Behandlungen durchführen.

### Spezifische Unterstützungsformen

Die Betroffenen berichteten davon, dass sie die MZEB insbesondere für Unterstützung in der Hilfs- und Heilmittelversorgung aufsuchten. Eine interdisziplinäre Hilfsmittelberatung konnte dazu dienen, die wiederkehrende Begründungspflicht für Heil- und Hilfsmittel gegenüber der jeweiligen Krankenkasse aufzufangen. Entsprechend leistete ein MZEB einen zentralen Beitrag bei der Durchsetzung von Hilfsmittelanträgen gegenüber der jeweiligen Krankenkasse durch die Formulierung von Anträgen, Widersprüchen und Begründungen. Mitarbeiter*innen begleiteten die Erprobung von Hilfsmitteln und koordinierten Termine mit Leistungserbringern. Die Bedeutung dieser Tätigkeit für die Betroffenen verdeutlicht die Aussage einer Patientin, die dies als extreme Entlastung beschrieb:

„Das musste ich früher ALLES alleine machen mit meiner Mama. Und jetzt hab ich < Physiotherapeutin MZEB > die das macht. … Aber ohne die hätte ich das alles nicht geschafft. Also es ist schon unheimlich gut, (.) da so eine Physiotherapeutin zu haben, die da ein bisschen Plan hat und sich in diese ganzen Fälle einsortiert und (.) macht und tut. … Das war so mein Engel und mein Halt in den zwei Jahren“ (A1Pw24_0011, Patient*in, Z. 399-400).

Diese Unterstützung bezog sich dabei nicht nur auf die fachliche Expertise der Mitarbeitenden und die koordinativen Leistungen, sondern auf die emotionale Begleitung und Durchsetzung der Selbstbestimmung und der Wünsche der Patientin.

Auch wenn die Unterstützung durch das MZEB geschätzt wurde, äußerte eine Teilnehmerin ihr Bedauern darüber, dass diese Unterstützung überhaupt notwendig sei. Sie sah sich als Kundin und Expertin ihrer eigenen Situation in der Lage, ihre Wünsche und Bedürfnisse konkret zu äußern. Dennoch misslang eine direkte Versorgung durch den Leistungserbringer:

„Obwohl es natürlich GUT ist, dass es jetzt/also die Möglichkeit gibt, dass man das dann in dem MZ/MZEB mit unterstützen lassen kann. (.) Umso mehr SCHADE finde ich es aber trotzdem, dass es nicht möglich ist, dass ich als Kundin, (.) hm ja, mit den Leuten da vom Sanitätshaus eine gute LÖSUNG finde“ (A1Pw35_0016, Patient*in, Z: 147-150).

Außerdem spielten Diagnostik und Begutachtung, beispielsweise im Zusammenhang mit der Förderung von Ausbildung oder Unterbringung in geeigneten Wohneinrichtungen, eine große Rolle. Einige Patient*innen erhielten die Verordnungen für Medikamente bzw. Zugang zu medikamentöser Therapie über das MZEB.

### Zeitliche, organisatorische und emotionale Entlastung

Einige Teilnehmende formulierten die konkrete Bestrebung, im MZEB einen festen Kreis behandelnder Ärzt*innen aufzubauen und eine möglichst umfassende Versorgung unter einem Dach zu erhalten. Dies wurde als eine zeitliche und organisatorische Entlastung erlebt, da mehrere Termine an einem Tag gebündelt werden könnten und sich der Koordinationsaufwand reduziere – was sich allerdings nur auf eines der beiden MZEB bezog. Insgesamt wurde bei der Versorgung im MZEB der Faktor Zeit als entscheidender Unterschied zur Regelversorgung und entsprechender Vorteil erlebt. Die Nutzer*innen erlebten durch mehr Zeit für Untersuchung, Begutachtung und Gespräch Wertschätzung und damit einen Raum, in dem sich Versorgende auf Bedarfe und Bedürfnisse einlassen konnten. Wurden eine dauerhafte Anbindung und Versorgung im MZEB möglich und von den Teilnehmenden gewünscht, wurde diese neu gewonnene Kontinuität in der Versorgung als eine positive Veränderung erlebt. Das MZEB war dabei der Ort, an dem alle Informationen über die betroffene Person vorlagen und den Behandler*innen bekannt oder zugänglich waren. Einige Teilnehmende beschrieben diese kontinuierliche Betreuung und die Beziehung zu den Versorgenden im MZEB als emotionale Entlastung. Der explizite Ausschluss von Behandlungen in einem MZEB führte dabei zu Irritationen. Während in der Regelversorgung eine mangelnde Bereitschaft und Expertise niedergelassener Ärzt*innen, Lösungen für individuelle Probleme zu finden, attestiert wurde, kann im Zusammenhang mit den MZEB eine gewisse Experimentierfreude, d. h. das Gehen neuer Wege in der Behandlung bzw. Versorgung, erkannt werden. Zentrale Aspekte schienen die Offenheit und Bereitschaft der Mitarbeitenden des MZEB zum „Ausprobieren“ zu sein.

### Veränderungspotenzial

MZEB wiesen das Potenzial auf, die Versorgung zu verändern, indem Zugänge zu Leistungen geschaffen, Leistungen erbracht oder angestoßen wurden. Dies konnten sowohl medizinische Leistungen als auch Leistungen der Teilhabeförderung betreffen. Zugang zu medizinischen Leistungen sahen wir in einem MZEB z. B. durch das direkte Erbringen von Leistungen, wie z. B. Botoxinjektionen, oder auch durch die Vermittlung an Leistungserbringer, wie ein Krankenhaus zur Röntgenaufnahme unter Narkose. Zugang zu teilhabefördernden Leistungen sahen wir z. B. im Rahmen von Verordnung und Umsetzung von Hilfsmittelversorgung. In anderen Fällen stellte eine umfassende und spezialisierte Diagnostik die Voraussetzung für den Zugang zu weiteren Behandlungs- und Unterstützungsleistungen dar, z. B. den Erhalt eines Grades der Behinderung (GdB).

MZEB haben neben der Veränderung der Versorgungsituation auch das Potenzial, die Wahrnehmung der Versorgung zu verändern. In einigen Fällen spielten Veränderungen des Gesundheitszustands, das Stellen einer Diagnose oder die Initiierung einer Behandlung in den Interviews zum 2. Erhebungszeitpunkt eine untergeordnete Rolle. Es wurde jedoch deutlich, dass sich die Wahrnehmung der Versorgung positiv verändert hat. Während im Rahmen der Regelversorgung das Gefühl dominierte, nicht ins Schema zu passen, eine „Sensation“ bzw. ein „Sonderfall“ zu sein und nicht verstanden zu werden, beschrieben einige Teilnehmer, sich im MZEB wertgeschätzt und in ihren Bedürfnissen ernstgenommen zu fühlen.

### Ambivalenzen der Nutzung von MZEB

Die Nutzung des MZEB ging auch mit Ambivalenzen und Widersprüchen einher. Einige Patient*innen bedauerten es, eine spezialisierte Einrichtung für ihren Versorgungsbedarf aufsuchen zu müssen, und die damit fehlende Teilhabe an Regelversorgung. Einzelne mieden das MZEB aus diesem Grund. Es gab jedoch auch diejenigen Teilnehmer*innen, die eine spezialisierte Versorgung mit dem Eindruck „hier bin ich normal“ begrüßten. Ebenso ambivalent wurde die Vereinigung mehrerer Fachdisziplinen in einem MZEB verhandelt: Auf der einen Seite waren Patient*innen, die hier eine Einschränkung ihrer freien Arztwahl wahrnahmen. Auf der anderen Seite waren diejenigen, die gern die Vorteile einer Einrichtung annahmen, in der „alles unter einem Dach“ ist, was auf eines der untersuchten MZEB zutraf. Ähnlich verhielt es sich mit dem Eindruck, Verantwortung an Fachpersonen abgeben zu können, im Gegensatz zu dem Gefühl, bevormundet zu werden und Einbußen in der Selbstbestimmung erdulden zu müssen. Zu guter Letzt löst die Parallelität von Versorgungsvorgängen, die im MZEB ggf. angestoßen wurden, entweder Erleichterung aus, weil nun endlich Bewegung in die Versorgung kommt. Oder aber dies führte zu Beklemmungen, weil es zu einem Dominoeffekt verschiedener zeitgleich ausgelöster Verordnungen und damit einer Kaskade von terminlichen und finanziellen Herausforderungen kam.

### Ungedeckte Bedarfe

Im Rahmen unserer Interviewstudie und bei den Beobachtungen wurden weitere ungedeckte Versorgungsbedarfe deutlich. Es handelt sich insbesondere um einen gravierenden Bedarf an psychologischer Beratung und Psychotherapie und an sozialrechtlicher Beratung, ein Bedarf an Informationen und Netzwerkmöglichkeiten, die über die medizinische Versorgung hinausgingen, ein Bedarf an Beratung zu den Themen Verhütung, Schwangerschaft und Kinderwunsch und einen Bedarf an Unterstützung bei der Suche nach einem Therapieplatz bzw. einer geeigneten (Fach‑)Klinik oder Rehaeinrichtung. Die Teilnehmenden zeigten teilweise große Ernüchterung bei der Erkenntnis, dass im MZEB selbst nach Feststellung des Bedarfes keine Therapie bzw. keine institutionsinterne Weiterleitung an Spezialist*innen erfolgen konnte.

### Rolle aus Sicht der Versorgenden

Aus Sicht der Versorgenden handelte es sich bei den MZEB aktuell v. a. um „Inseln“ der Spezialisierung, in denen „Vorreiter“ Pionierarbeit leisteten. Sie sahen sich als Netzwerker*innen, die Hausärzt*innen bei der Versorgung von Patient*innen mit komplexen Bedarfen entlasten können. Die Leistungen der MZEB waren durch die vielfältigen Bedarfe der Patient*innen breit gefächert. Sie erstreckten sich über hochspezialisierte medizinische Versorgung (in einem MZEB) bis weit in sozialmedizinische Handlungsfelder und sie wiesen Schnittstellen zu Einrichtungen der Eingliederungshilfe auf.

Beide teilnehmenden MZEB unterschieden sich im Schwerpunkt, in der Größe, Struktur und ihrer Historie erheblich. Die Rollen, die sie einnehmen konnten, hingen maßgeblich von der Ausgestaltung der jeweils bundeslandspezifischen Verträge ab. Der Vernetzung der MZEB untereinander und der Schaffung eines breiten und zugänglichen Angebots für die Vielzahl heterogener Patient*innen kam eine hohe Bedeutung zu.

## Diskussion

Die Ergebnisse der MeZEB-Studie ermöglichen einen differenzierten Blick auf die Versorgungsrealität der MZEB und ihre potenzielle Rolle im Versorgungssystem. Über allem steht der Begriff der Heterogenität. Bereits die beiden betrachteten MZEB unterschieden sich hinsichtlich ihrer Arbeitsweise so grundlegend, dass für Betroffene und Angehörige kaum vorhersehbar ist, welche Art der Versorgung sie unter dem Dach des MZEB erwarten können. Dies hängt unter anderem mit den unterschiedlichen institutionellen Traditionen der Einrichtungen zusammen. Zugleich werden MZEB maßgeblich durch das Engagement, die Expertise und die Erfahrungen der beteiligten Akteur*innen geprägt, die ihre Gründung häufig erst ermöglichen. Da einzelne MZEB jedoch nur einen begrenzten Ausschnitt der Bedarfe von Menschen mit einem umschriebenen Behinderungsspektrum abdecken können, bleiben ihre Angebote zwangsläufig selektiv. Gerade die fehlende Möglichkeit zur Behandlung in einem der beiden MZEB hat für große Irritationen und Ernüchterung gesorgt. Deutlich wurde auch, dass die MZEB sehr unterschiedlich und aus sehr divergierenden Motivationen heraus genutzt wurden. Dabei geben sie offensichtlich nur für einen Teil der Betroffenen einen angemessenen Versorgungsrahmen ab, der für den anderen Teil zugleich eine qualitative Verbesserung ihrer Versorgungssituation darstellte.

Während die UN-BRK spezielle Gesundheitsleistungen einfordert, die so gemeindenah wie möglich zur Verfügung stehen sollten, erscheint die Heterogenität der MZEB eher geeignet, als Anlaufstelle für Betroffene mit bestimmten Beeinträchtigungen aus einer sehr großen Region dienen zu können. Eine zentrale Herausforderung wird sein, dass sich die unterschiedlichen MZEB am regionalen Versorgungsbedarf orientieren sollten. Es ist sinnvoll, in größeren Versorgungsregionen zu denken, in denen möglichst viele fachliche Schwerpunkte in arbeitsteiliger Zusammenarbeit abgedeckt werden [6]. Zugleich sollten die einzelnen MZEB in die Lage versetzt werden, als erste spezialisierte Anlaufstelle die Versorgung in Zusammenarbeit mit regionalen Versorgungsstrukturen und anderen MZEB zu koordinieren sowie neben einer bedarfsgerechten Diagnostik auch geeignete Behandlungsangebote vorzuhalten. Dazu gehören die bislang als deutlich defizitär wahrgenommene psychotherapeutische (vgl. [13, 14]) und auch gynäkologische Versorgung bzw. Leistungen bei Kinderwunsch, Schwangerschaft und Mutterschaft.

### Limitationen

Qualitative Fallstudien sind grundsätzlich dadurch begrenzt, dass generalisierende Aussagen nur eingeschränkt möglich sind und in erster Linie argumentativ begründet werden müssen. Die zentrale Beobachtung dieser Studie – die ausgeprägte Heterogenität hinsichtlich Versorgungsangeboten, Erwartungen und Bedürfnissen sowie der fachlichen Schwerpunkte und Kompetenzen der MZEB – wurde bereits theoretisch in der Einleitung hergleitet und in der kontrastierenden Analyse der beiden MZEB deutlich bestätigt. Vor diesem Hintergrund spricht vieles für die Annahme, dass Heterogenität ein grundlegendes Merkmal der MZEB-Landschaft darstellt. Eine belastbare Überprüfung dieser Annahme würde jedoch eine repräsentative Untersuchung erfordern, die allerdings aufgrund ihres breiteren Zuschnitts voraussichtlich weniger tiefgehende Einblicke bieten würde.

Erstmals wurde eine größere Gruppe gezielt ausgewählter Nutzer*innen von MZEB im Rahmen von offenen Leitfadeninterviews zu ihren Versorgungserfahrungen befragt. Aufgrund der begrenzten Wortzahl konnten wir in diesem Beitrag allerdings nur sehr bedingt Belege in Form von Zitaten aufführen. Gerade die nur sehr eingeschränkte Transparenz des Forschungsprozesses, die nicht weiter ausgeführte Begründung methodischer Entscheidungen sowie die begrenzenden Möglichkeiten einer angemessen empirischen Verankerung der Aussagen im vorliegenden Publikationsformat stellen substanzielle Einschränkungen dar. Einzelne methodisch relevante Schritte, wie Entscheidungen zum Sampling, eine klinisch orientierte Merkmalsdarstellung der Befragten bzw. der Patient*innen oder das Vorgehen bei der Zusammenführung von Daten aus unterschiedlichen Quellen konnten nicht angemessen dargelegt und in ihren möglichen Bedeutungen für die Aussagekraft der Studie diskutiert werden. Weiterführende Informationen und Diskussionspunkte finden sich im genannten Abschlussbericht für den Innovationsfonds [12].

## Fazit

Es bleibt festzuhalten, dass die Rolle der MZEB in der Gesundheitsversorgung – positiv formuliert – sehr vielfältig, negativ formuliert jedoch noch weitgehend unbestimmt ist. Eine stärkere Orientierung an regionalen Versorgungsbedarfen erscheint geboten. Ebenso sind sowohl die Möglichkeit der Behandlung als auch die Notwendigkeit der Kooperation zwischen den MZEB weiterzuentwickeln, um den Nutzen ihrer Spezialisierungen zu erhalten. Die MZEB sollten letztlich Kompetenzzentren in ihrer Region darstellen, die auch Verantwortung in der Aus‑, Fort- und Weiterbildung übernehmen. Weitere Schlussfolgerungen aus dem Projekt in Form von Handlungsempfehlungen, die zusätzlich auf der Diskussion mit verschiedenen Stakeholdergruppen basieren, finden sich bei Weiß et al. [12].

## Data Availability

Die während der vorliegenden Studie erzeugten und/oder analysierten Datensätze sind nicht öffentlich zugänglich, da sie aufgrund der enthaltenen vielschichtigen Informationen (Interviewtranskripte, Beobachtungsprotokolle) mögliche Rückschlüsse auf die Teilnehmenden zulassen und damit die Anonymität der Teilnehmenden nicht gewährleistet wäre. Auf begründete Anfrage können nach Absprache und entsprechender Prüfung zur Anonymität Ausschnitte aus dem Material angefordert werden.
